# Comparative assessment between objective and subjective methods in slides stained by immunohistochemistry

**DOI:** 10.5935/1808-8694.20130129

**Published:** 2015-10-08

**Authors:** Fernando de Andrade Quintanilha Ribeiro, Celina Siqueira Barbosa Pereira, Ricardo Junchen Chi, Patrícia Lumi Yokomizo, José Humberto Tavares Guerreiro Fregnani, Rafael Malagoli Rocha

**Affiliations:** a PhD; Professor, Department of Otorhinolaryngology, School of Medical Sciences of Santa Casa de São Paulo; b PhD; Adjunct Professor, Department of Morphology, School of Medical Sciences of Santa Casa de São Paulo; c 6^th^-year Medical Student, School of Medical of Sciences of Santa Casa de São Paulo; Resident Physician; d 3^rd^-year Medical Student, School of Medical Sciences of Santa Casa de São Paulo; e PhD, Researcher at the Researcher Support Core of the Institute of Education and Research of the Barretos Cancer Hospital; f PhD, Research Scientist, Department of Pathology, AC Camargo Hospital - São Paulo. School of Medical Sciences of the Santa Casa de São Paulo

**Keywords:** biomedical technology evaluation, cholesteatoma, compared histology, immunohistochemistry

## Abstract

Objective methods of assessment are often required in scientific studies. Histological tests with immunohistochemical staining can be assessed by photometry.

**Objective:**

To compare this objective method with the subjective evaluation performed by three independent examiners, using slides of acquired middle ear cholesteatomas.

**Method:**

We selected a total of 54 cholesteatoma images, immunohistochemically stained by anti-TNF-R2 (32 slides) and anti-TGF-α, (22 slides). The secondary antibody used in the two groups was the Max Polymer Detection System (Novo Link Kit, Novocastra®, UK). The samples were processed by a digital slide scanner (ScanScope - Aperio). The selected sites were analyzed by photometry.

**Results:**

The objective assessment by photometry was compared with the subjective evaluation by three examiners and subjected to statistical analysis. The Statistical analysis revealed moderate reproducibility (K values between 0.41 and 0.60) for both groups.

**Conclusion:**

Our study showed that the irregular characteristics of middle ear cholesteatoma slides stained by immunohistochemistry prevents its proper objective evaluation, while the subjective assessment by experienced examiners was more reliable.

## INTRODUCTION

Many journals have requested the authors to use an objective method for the evaluation of histological sections, both in the usual stains of hematoxylin-eosin and immunohistochemistry. In the latter, the reaction is observed by viewing a brownish stain pervading specific parts of the cell, such as the cell membrane, the cytoplasm or the nucleus1., 2., 3.. By analyzing this expression, an experienced examiner can classify it in a qualitative (present or absent) and quantitative (weak, moderate, severe) manner. Since this evaluation is subjective, often times it requires more than one examiner for the quantitative analysis of the material. Several objective methods for analyzing these reactions have been proposed4., 5., 6., 7..

Photometry is one of these methods, and it consists of scanning the slide and analyzing the image, using a software that reads the light flow, that is, the greater the impregnation of the tissue by the immunohistochemical reaction, the lower the value observed at the photometer. However, various tissues are heterogeneous when submitted to immunohistochemical analysis, with areas where the reaction is intense and others with weak or moderate antigen expression. In these cases, the examiner classifies the fragment based on the predominant staining in the field studied.

The objective of this study is to compare the reproducibility between the subjective and objective methods to evaluate the expression of immunohistochemical reactions in the matrix of acquired middle ear cholesteatoma in humans.

## METHOD

This paper was submitted for review and approval by the Ethics Committee of the Institution (project # 163/11).

We used 54 slides previously stained by immunohistochemistry in the Department of Morphology of the institution. All samples were fixed in 10% formaldehyde and processed by the usual techniques, embedded in paraffin. The three micrometer-thick slices were obtained by a rotary micrometer and previously evaluated with hematoxylin-eosin (HE) to confirm matrix (epithelium) and perimatrix presence and integrity of the. For immunohistochemistry, we promoted the blocking of endogenous peroxidase and subsequently the antigen recovery with citrate (pH 6.0) in a steamer. The primary antibodies used were: anti-TNF-R2 and anti-TGF-α (LabVision®, USA) in the 1:100 titer. The primary antibody used in 32 slides was the TNF-R2 (group 1), while the TGF-α was used in the other 22 slides (group 2). The secondary antibody used in the two groups was the Max Polymer Detection System (Novo Link Kit, Novocastra®, UK).

The slides were analyzed with an Axioscope 40 (Carl Zeiss) optical microscope model with a 10x eyepiece and 10x, 20x and 40x objective lens. The selection of two groups of slides stained with different primary antibody was a way to better assess any changes in cytoplasm, membrane and nucleus staining. In group 1, the anti-TNF-R2 antibody response was cytoplasmic and nuclear, and in group 2, the anti-TGF-α antibody stained only the cell cytoplasm. The immunohistochemical reaction subjective evaluation was performed by three experienced examiners, independently, qualitatively (present or absent) and quantitatively, according to staining intensity (weak = 1, moderate = 2 and severe = 3). When there was no agreement between the three assessments, the slide was discussed, seeking a consensus.

These same samples were processed by a ScanScope histological slides scanner (Aperio), at the Department of Pathology, obtaining high-resolution digital images of the histological sections. The images were projected on the monitor screen and, by means of a digital marking tool, we defined the most representative area of the immunohistochemical reaction on each slide. The selected areas were analyzed by photometry for the objective assessment of their optical density.

The device evaluated the optical transparence of each histological section by assigning a number that corresponded to the average intensity of the immunohistochemical staining of the selected area (Iavg) obtained by counting pixels. The Iavg (Average Intensity of all pixels) is a parameter that measures the brightness of a pixel. The Iavg scale is graded from zero (black) to 255 (white), so that high Iavg values indicate clearer and brighter images. This occurs because the pixel brightness is proportional to the amount of light passing through the slide in the scanner.

For statistical analysis purposes, it was necessary to categorize the expression of the objective analysis markers in three groups (weak, moderate and strong expression), to compare it with the subjective evaluation and determine whether there was reproducibility between the methods. We calculated the range of values (difference between the minimum and maximum values) for each group of slides. The value obtained was divided into three parts. The first third, with the lowest values corresponded to the grade 3 class (strong expression). The second third corresponded to grade 2 (moderate expression) and the last third corresponded to Grade 1 (weak expression). After turning numeric variables into ordinal numbers, we used the weighted *kappa* test *(K)*.

The study was double blinded, since the examiners who obtained responses by the subjective method were not aware of the objective assessment outcomes, and vice versa.

## RESULTS

Considering the objective evaluation, the lavg interval obtained for the first group of slides stained with anti-TNFR2 was 98.97 to 183.78. The range between the maximum and minimum values was 84.81. Dividing this by 3, we found 28.27. Thus, the amplitude ranges were: 98.97 to 127.23 (grade 3), 127.24 to 155.51 (grade 2) and 155.52 to 183.78 (grade 1). The Iavg value is inversely proportional to the grade numbering, in other words, a low intensity interpreted by the examiner (subjective evaluation) corresponds to a high Iavg value (objective evaluation) obtained by photometry.

For the second group of slides stained with the anti-TGF-α antibody, the lavg range was from 114.61 to 194.28. The range between the maximum and minimum values was 79.67. Dividing this by 3, we find 26.55. Thus, the amplitude ranges were 114.61–141.16 (grade 3), 141.17 to 167.72 (grade 2) and 167.73 to 194.28 (grade 1).

The subjective and objective evaluations of the slides from the two groups, and the lavg value in pixels found for the area marked on each slide, are shown in Tables 1 and 2.

**Table 1:** Subjective evaluations, Iavg in pixels and objective assessments of the intensities of immunohistochemical reactions of the group of slides stained with anti-TNF-R2.

Slides	Subjective assessment	Iavg	Collective assessment	Slides	Subjective assessment	Iavg	Objective assessment
1	1	159.341	1	17	3	131.028	2
2	1	159.406	1	18	3	124.077	3
3	3	140.696	2	19	2	128.979	2
4	2	122.121	3	20	2	115.774	3
5	1	151.858	2	21	2	159.835	1
6	1	176.853	1	22	1	180.072	1
7	2	160.285	1	23	1	166.226	1
8	2	156.416	1	24	2	128.861	2
9	2	152.165	2	25	1	170.386	1
10	2	165.712	1	26	2	148.767	2
11	1	166.643	1	27	2	160.342	1
12	2	163.404	1	28	3	98.9718	3
13	1	183.777	1	29	1	154.774	2
14	1	165.834	1	30	3	110.368	3
15	2	149.73	2	31	1	161.868	1
16	3	137.216	2	32	2	142.923	2

**Table 2:** Subjective assessments, lavg in pixels and objective assessments of the intensities of immunohistochemical reactions in the group of slides stained with anti-TGF-α.

Slides	Subjective assessment	Iavg	Objective assessment	Slides	Subjective assessment	Iavg	Objective assessment
1	2	167.89	1	12	2	114.61	3
2	3	118.61	3	13	1	125.79	3
3	3	127.36	3	14	3	156.89	2
4	1	155.02	2	15	1	175.69	1
5	1	167.70	2	16	2	161.34	2
6	1	174.55	1	17	2	161.65	2
7	2	143.58	2	18	3	153.83	2
8	1	155.10	2	19	3	131.46	3
9	2	137.99	3	20	1	194.28	1
10	3	116.41	3	21	2	164.89	2
11	3	128.73	3	22	3	156.85	2

When comparing the results of the two assessments for the groups studied, the percentage of accuracy between observers and photometry was 59.4 % for group 1 and 54.5 % for group 2, values below what had been expected (Tables 3 and 4).

**Table 3:** Comparison between subjective and objective assessments of cases in group 1.

Slide	Subjective	Objective	Slide	Subjective	Objective
1	1	1	17	3	2
2	1	1	18	3	3
3	3	2	19	2	2
4	2	3	20	2	3
5	1	2	21	2	1
6	1	1	22	1	1
7	2	1	23	1	1
8	2	1	24	2	2
9	2	2	25	1	1
10	2	1	26	2	2
11	1	1	27	2	1
12	2	1	28	3	3
13	1	1	29	1	2
14	1	1	30	3	3
15	2	2	31	1	1
16	3	2	32	2	2

**Table 4:** Comparison between subjective and objective assessments of those in group 2.

Slide	Subjective	Objective	Slide	Subjective	Objective
1	2	1	12	2	3
2	3	3	13	1	3
3	3	3	14	3	2
4	1	2	15	1	1
5	1	2	16	2	2
6	1	1	17	2	2
7	2	2	18	3	2
8	1	2	19	3	3
9	2	3	20	1	1
10	3	3	21	2	2
11	3	3	22	3	2

After turning the objective evaluation values into ordinal numbers, it can be compared with the subjective evaluation by the weighted *kappa (K)* method, which allows the calculation of the reproducibility between two variables when they are ordinals. The *K* value for the first group was 0.48 and for the second group, 0.41. The statistical analysis revealed moderate reproducibility (K values between 0.41 and 0.60) between subjective and objective analyses.

## DISCUSSION

Several studies have been led demonstrating the usefulness of objective methods in diagnostic analysis of histological sections. The development of new evaluation techniques aims at making the analysis more objective and standardized, creating a reliable and reproducible model.

Objective methods have been described for evaluating cell impregnations using immunohistochemistry6., 7.. However, we did not find references in the literature concerning the use of these methods to investigate acquired middle ear cholesteatomas in humans.

We used two lots of slides with different staining characteristics to provide the study with greater reliability. When we compared the percentage of matching results between the subjective and objective assessments in both groups, the values fell between 50% and 60%. The reproducibility calculated using the *Kappa* method was moderate, with values at the lower limit within the range. For a new method to be used as a substitute for another already established, the reproducibility should be much better than that found in the present study, with *Kappa* values from 0.81 to 1.00 (very good reproducibility).

**Figure 1:**
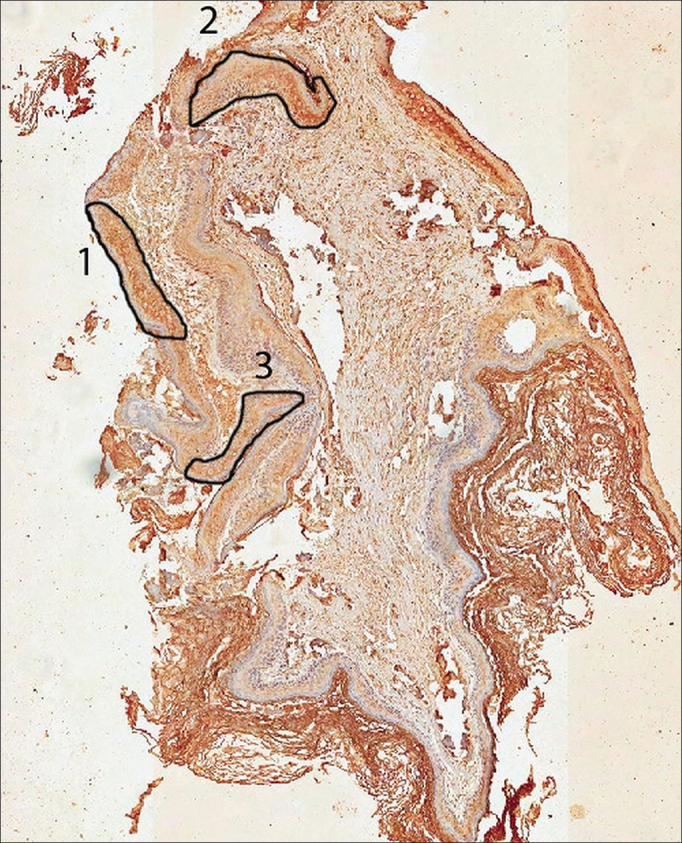
Scanned slide submitted to markings in three areas according to the antibody reaction intensity (group 1). Area 1 was considered representative of the case and areas 2 and 3 were disregarded.

One possible explanation for this discrepancy in results is the lack of reaction uniformity in histological sections of middle ear cholesteatomas. Since the staining is not homogeneous, being different in different areas of a single slide, it is necessary to select the most representative region of the slide to undergo analysis in the photometer, which in itself reduces the method's objectivity (Figure 1). The great diversity of histological structures' components can also impact the objective assessment.

A trained examiner can consider the entire tissue being studied, irrespective of variations found in each slide, and choose the most suitable area of the histological section to set the intensity of the immunohistochemical reaction, which is still very limited in photometry.

Thus, although objective diagnostic methods are increasingly being used, their employability in the evaluation of immunohistochemical expression in cases of acquired cholesteatomas of the middle ear with these antibodies was questionable.

We conclude that human experience and personal judgement make a more accurate assessment by considering, in the entire slide, the epithelium thickness, the cytoplasmic or nuclear impregnation, technical artifacts, etc. In photometry we would have to choose multiple locations on the slide, or even evaluate parts of the cell and average out the results to obtain outcomes similar to those achieved by a good histologist taking a look.

## CONCLUSION

The present study showed a moderate reproducibility between the objective method (photometry) and the subjective analysis of immunohistochemical reactions in fragments of acquired human middle ear cholesteatoma epithelium.
